# Comparative Plasma Exposure and Lung Distribution of Two Human Use Commercial Azithromycin Formulations Assessed in Murine Model: A Preclinical Study

**DOI:** 10.1155/2013/392010

**Published:** 2013-08-29

**Authors:** Virginia Rivulgo, Mónica Sparo, Mónica Ceci, Elida Fumuso, Alejandra Confalonieri, Gastón Delpech, Sergio F. Sánchez Bruni

**Affiliations:** ^1^Laboratory of Pharmacology, Faculty of Veterinary Medicine, Universidad Nacional del Centro de la Provincia de Buenos Aires, Paraje Arroyo Seco s/n., 7000 Tandil, Argentina; ^2^Consejo Nacional de Investigaciones Científicas y Tecnológicas (CONICET), Argentina; ^3^Tandil Veterinary Research Center (CIVETAN-CONICET), Argentina

## Abstract

Azithromycin (AZM) therapeutic failure and relapses of patients treated with generic formulations have been observed in clinical practice. The main goal of this research was to compare in a preclinical study the serum exposure and lung tissue concentration of two commercial formulations AZM-based in murine model. The current study involved 264 healthy Balb-C. Mice were divided into two groups (*n* = 44): animals of Group A (reference formulation -R-) were orally treated with AZM suspension at 10 mg/kg of b.w. Experimental animals of Group B (generic formulation -G-) received identical treatment than Group A with a generic formulation AZM-based. The study was repeated twice as Phase II and III. Serum and lung tissue samples were taken 24 h post treatment. Validated microbiological assay was used to determine the serum pharmacokinetic and lung distribution of AZM. After the pharmacokinetic analysis was observed, a similar serum exposure for both formulations of AZM assayed. In contrast, statistical differences (*P* < 0.001) were obtained after comparing the concentrations of both formulations in lung tissue, being the values obtained for AUC and Cmax (AZM-R-) +1586 and 122%, respectively, than those obtained for AZM-G- in lung. These differences may indicate large differences on the distribution process of both formulations, which may explain the lack of efficacy/therapeutic failure observed on clinical practice.

## 1. Introduction

**Figure 1:**
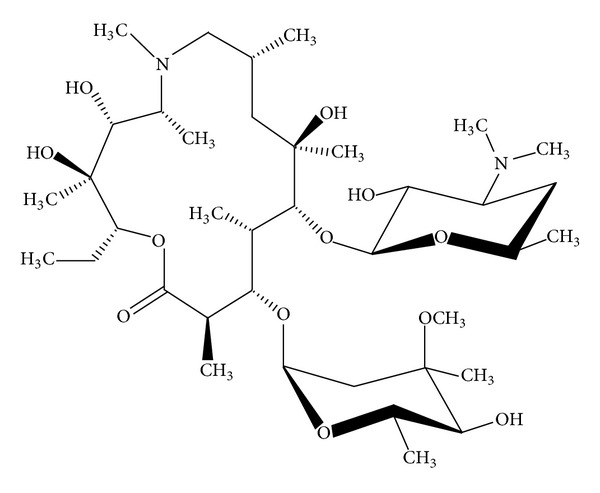
Chemical structure of Azithromycin.

Azithromycin (AZM) is a macrolide antibiotic that chemically differs from erythromycin by methyl-substituted nitrogen atom at position 9a in the macrolide ring. AZM is composed by fifteen-membered ring structure having two sugar moieties, several hydroxyl groups, two tertiary amino groups, and one oxycarbonyl group (Figure 1). It is only slightly soluble in water but highly soluble in lower alcohols and yet shows low viscosity in these solvents. These chemical modifications derived from the primary molecule (erythromycin) have produced profound modifications on the AZM *in vitro* spectrum, potency, and superior stability to acid environment [1]. *In vivo* studies demonstrated that AZM concentrations are highly accumulated in alveolar macrophages and lung being 100% higher than those reported in plasma [1, 2] by which this has largely been recommended for the treatment of some respiratory diseases, sexually transmitted diseases, some skin diseases, and otitis media. Among other reasons, this antibiotic formulated mainly as suspension or tablets is used in human and in veterinary medicine.

The need of decreasing the cost of the treatments moves the replacing of bioequivalent innovator drugs with generic drugs. Around the world, the replacement is promoted in some case of disease when the generic drugs are bioequivalent with his respective innovator drugs [3, 4]. However, therapeutic failure and adverse effects in the use of generic drugs in humans in specific diseases like antiepileptic drugs have been reported [4, 5].

Interestingly, AZM was recently reported as a new chiral selector in capillary electrophoresis studies, having multiple stereogenic centers [6]. The presence of multiple chiral centers and different functional groups may undergo multiple interactions with the analyte enantiomeric molecules for chiral recognition in a similar way to other macrocyclic antibiotic chiral selectors [6]. 

In recent years, the drugs stereochemistry became in a significant issue for the pharmaceutical industry. The stereoisomers interact differently with the macromolecules in the body, while waiting for the process of passive diffusion/transporters uptake into cell membranes which is equivalent for both moieties [7–9]. Thus, differences in active transport in serum secretion protein-binding, metabolism, and pharmacological effects for both molecules may have differences for achieving the biophase. Such steroisomers frequently differ in terms of their biological activity and pharmacokinetic (PK) profiles and the use of such mixtures contributes to the adverse effects of the drug particularly if they are associated with the inactive or less active isomer [10, 11].

Most drugs and drug-like molecules are likely to bind to multiple transporters, for example; drugs are known to interact with no fewer than six targets and many proteins are known to interact with hundreds of drugs to get the biophase [12]. However, also it is worth to mention the interaction drug-drug with proteins, such as transporters, rather than phospholipids, becoming on the area of adverse effects [12]. 

Therapeutic failure and relapses of patients treated with generic formulations have been observed in some hospitals [13, 14]. However, those products were approved for commercialization, based on pharmacokinetic/bioequivalence studies. Similar clinical observations were seen in veterinary clinical practice using generic human formulations in *Rhodococcus equi* pneumonia diagnosed foals. For that reason, we hypothesized that the lung distribution of the active principle (API) and its consequent efficacy of two bioequivalent formulations AZM-based are different.

The inconvenience of assessing AZM in lung parenchyma via biopsy in live mammals is derived in the current study. The main goal of this research was to compare in a pre-clinical study the AZM serum exposure and lung tissue concentration of two commercial formulations AZM-based in murine model. 

## 2. Materials and Methods

### 2.1. Bacterial Strains

Bacterial strains used were: *Kocuria rhizophila* (ex-*Micrococcus luteus*) ATCC 9341 an azithromycin susceptible strain.

### 2.2. Experimental Design

The current study involved 264 healthy Balb-C mice weighing 18–20 g. Mice were normally fed having free access to water. The experiment was designed using 88 experimental animals (Phase I) and repeated twice (Phases II and III). For each experimental Phase, Balb-C mice were divided in two groups (*n* = 44): animals of Group A (reference formulation -R-) were orally treated (via cannula) with AZM suspension at 10 mg/kg of body weight (Zithromax, Pfizer, Argentina). Experimental animals of Group B (generic formulation -G-) received identical treatment to that of Group A with a generic formulation AZM-based (Azitromicina Richet, Richet S.A, Argentina).

The whole experiment was performed under the policies of Animal Welfare of UNCPBA Veterinary Faculty (http://www.vet.unicen.edu.ar).

Experimental animals were humanely euthanized. Serum and lung tissue samples were taken as follows: time 0 (pre treatment) and after treatment at 0.5, 1, 1.5, 2, 4, 6, 8, 10, 12, and 24 h. Each sampling timepoint per experimental phase is represented by 4 animals. After samples collection, the blood was centrifuged at 1.5 ×g by 10 min and the serum was stored at −80°C, until analysis. Lung samples were taken and wrapped in aluminium paper upon being stored at −80°C. 

### 2.3. Microbiological Assay

The concentration of azithromycin (AZM) was measured in serum and lung tissue using the microbiological assay, according to Breier et al. [15]. The strain *Kocuria rhizophila* (ex-*Micrococcus luteus*) ATCC 9341 was used as the indicator. Briefly, an aliquot (200 *μ*L) of *Kokuria rhizophila *was inoculated in 3 mL of brain-heart infusion (BHI) broth and incubated at 35°C for 24 h. Afterwards, a suspension with a viable count of 1 × 10^8^ CFU/mL was prepared; 200 *μ*L of this suspension was added to a tempered tube containing 7 mL of soft-agar Mueller Hinton (Lab. Britania; Argentina) and distributed evenly in Petri dishes with 13 mL of Mueller Hinton agar (Lab. Britania; Argentina, 15.5 g/L). The dishes were allowed to solidify and wells were punched and filled with 100 *μ*L from each serum sample. In lung tissue, each one was weighted (0.01 g) and emulsified in 1 mL of sterile distilled water in a glass-mortar, the resulting homogenate was sonicated and 100 *μ*L was placed in the respective well. Agar dishes were incubated for 24 hours at 35°C. A standard curve was done with a solution of AZM (Pfizer, US) between 0.025–5 *μ*g/mL for serum and 0.150 to 150 *μ*g/g for lung tissue samples. The results shown, represent the average of three experiments.

### 2.4. Statistical Analysis

Statistical analysis was performed using the nonparametric Mann-Whitney test, after comparing the results of the 3 phases of study.

### 2.5. Pharmacokinetic Analysis

The concentration versus time curves for AZM in serum and lung tissue obtained from each individual experimental animal after oral treatment with AZM was fitted by a PK Solution Software package (Summit Research Services, Ashland, OH, USA). Serum and lung tissue concentrations best fitted to a biexponential curve (*r* ^2^ = 0.9880 and *r* ^2^ = 0.998). The maximum concentrations (*C*_max⁡_) and time to *C*_max⁡_ (*T*_max⁡_) of AZM in serum and lung after oral administration were extrapolated from the plotted concentration time curve as the average of 4 animals per point/phase (12 animals average per point). Trapezoid AUCs were calculated according to Gibaldi and Perrier [16].

## 3. Results and Discussion

### 3.1. Microbiological Assay

 A bioassay method was used in this study to determine the concentrations of AZM serum and lung compartments after a single-dose oral administration. Since the microbiological assay seems to measure the total microbiological action, it was considered the more appropriate to measure the *in vivo* drug concentrations effectiveness, with the goal of correlating with the clinical and bacteriological cure observed on the clinical practice [15].

The assay was linear between 0.025 and 5.0 *μ*g/mL for serum and 0.150 and 150 *μ*g/g for lung tissue samples. Calibration curves yielded a correlation coefficient of 0.998 and 0.987 for serum and lung, respectively. It was assessed by assaying samples of murine serum and lung tissue sample, at three different concentrations, during the same day and three different days under the same experimental conditions. Intra-assay and interassay coefficients of correlation were lower than 3%. 

### 3.2. Pharmacokinetic in Serum and Lung Tissue Distribution Studies

**Figure 2:**
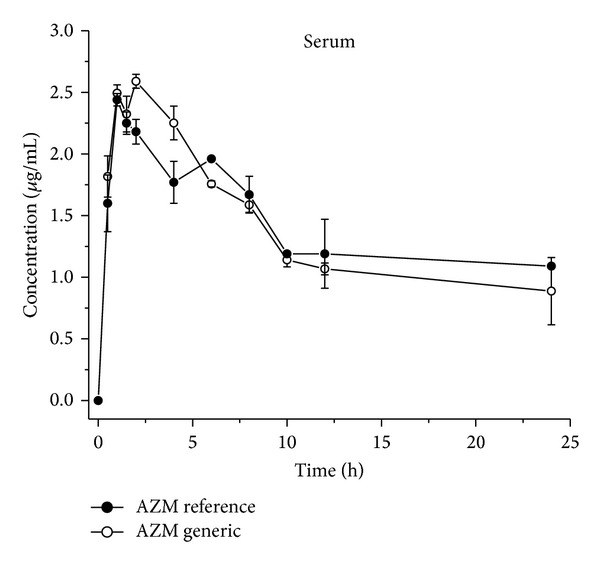
Comparative serum exposure concentrations (Mean ± SD) of two commercial formulations Azithromycin-based after given orally at 10 mg/kg as suspension in mice. Each point plotted in the curve is the average of 12 experimental animals.

**Figure 3:**
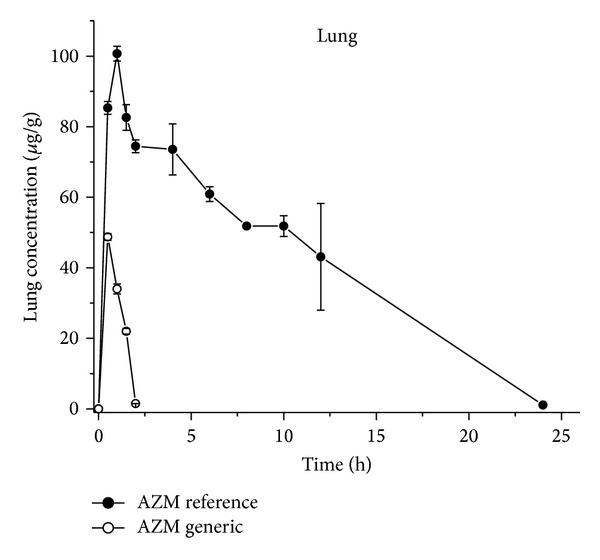
Comparative lung tissue concentrations of two commercial formulations Azithromycin-based after given orally at 10 mg/kg as suspension in mice: each point plotted in the curve is the average of 12 experimental animals.

After analysis of primary PK parameters (Table 1) was observed, a similar serum exposure for the two formulations of AZM assayed based in similar AUC values (AUC = 34.5 *μ*g·h/mL -AZM-R- and 33.3 *μ*g·h/mL -AZM-G-). No differences in *C*_max⁡_ and *T*_max⁡_ were observed in serum after both treatments (Table 1 and Figure 2). These outcomes indicated that the absorption process after both treatments was not affected. In contrast, statistical differences *P* < 0.001 were obtained after comparing the concentrations of both formulations in lung tissue. Thus, the values obtained for AUC and *C*_max⁡_ for the AZM-R- formulation increase by more 1586 and 122%, respectively, than those values obtained for AZM-G-formulation. The latter also correlates with the longer half life of elimination obtained for AZM-R (3.78 h) when compared with the AZM-G formulation (0.22 h). These statistical differences on the PK parameters reported in Table 1 and Figure 3 may indicate large differences on the distribution process of both formulations, which may explain the lack of efficacy/therapeutic failure observed on the clinical practice for the AZM-G-formulation. 

Macrolide antibiotics like AZM and Clarithromycin (CLR) are large molecular weight compounds and are substrates for apically polarized efflux transporters such as P-glycoprotein, which can potentially restrict intestinal absorption. However, despite these undesired physicochemical and biopharmaceutical properties, AZM and CLR exhibit moderate to excellent p.o. bioavailability in preclinical species and humans. Intestinal uptake transporters, such as organic anion transporting polypeptides (OATPs), can facilitate the uptake of drugs that are substrates and hence increase p.o. absorption [17].

It is pivotal to recognize some of the factors than it can alter the outcome of PK studies and therefore potentially altering the pharmacological response. When compared the serum exposure of AZM-R- with AZM-G-, neither PK differences nor absorption process was observed after the racemic formulation administration (Table 1, Figure 2). The AUC values and the similar displaying curves were obtained, letting us hypothesize that, specifically, AZM could not exhibit enantio-selective absorption when probably a chiral- imbalance is regarded with the API of the formulation. 

AZM, is highly distributed from serum to lung epithelium lining fluid (ELF), the infection site of pathogens. Transporter(s) expressed on lung epithelial cells may contribute to the distribution of the compounds to the ELF. The mechanisms of macrolides distribution are not still well known. However, Togami et al. [9] reported that AZM, Clarithromycin and Telithromycin, antibiotics are transported from plasma to ELF by MDR1 of lung epithelial cells. 

The significance of the stereochemical consideration in the area of antimicrobial agents is relevant when the alteration of any of the chiral centres results in a marked or total loss of activity [10, 12]. As reported previously, AZM was recently characterized as a new chiral selector with many stereogenic centers and different functional groups (like other macrolides, glycopeptides, ansamycins, and aminoglycosides), which allow for multiple interactions with the analyte enantiomeric molecules, resolving a wide range of enantiomers with very high selectivities [6]. These may explain the difference on the distribution process of both formulations assayed. The large difference obtained in favor of AZM-R- (+1586 and 122% for AUC and *C*_max⁡_, resp.) may be due by the selective binding to a “balanced chiral” AZM formulation to the transporters towards the lung tissue (biophase) (Figure 3). 

The promiscuous binding of pharmaceutical drugs and the transporter-mediated uptake into cells was recently described by Kell et al. [12]. Probably in a racemic generic formulation of multiple chiral molecules (as AZM), a minimal deficiency on the manufacture process may cause a chiral imbalance of the API with the consequent difference on distribution (transporters-mediated uptake into cells) and antimicrobial activity. Specifically the fit of the enantiomers to the different transporters/and receptor surface may be different and the binding energies of the interaction may also differ.

Reports by Zuluaga et al. [14] and Vesga et al. [18] demonstrated *in vivo* the no therapeutic equivalence of some generic drugs using multiple chiral antibiotics like vancomycin and gentamicin, in murine model. Interestingly those authors, based on an article reported by Henderson and Esham [19], stated that to approve generic versions of innovator (reference) products, comparative preclinical or clinical safety and/or efficacy studies are not required, assuming that generics, by pharmaceutical equivalence or bioequivalence studies, would generate similar outcomes to those obtained with the innovator API. 

## 4. Conclusions

In this paper have been assessed the serum exposure and lung distribution of two commercial AZM formulations, in a pre-clinical study in a healthy mice validated model. Differences on the absorption process were not found, however; marked modifications on the distribution process were eloquent. The results obtained in this trial could partially explain the lack of efficacy of some generic formulations AZM-based. Since identical serum concentrations do not reflect the AZM tissue distribution when compared AZM-R- with a generic formulation, perhaps drug regulatory agencies need to discuss this issue to complement the bioequivalence studies with a tissue distribution trial in a validated primary species (mice) model. 

## Figures and Tables

**Table 1:** Comparative pharmacokinetic parameters of serum and lung of both formulations azithromycin-based assayed.

PK parameter	AZM reference	AZM generic
Serum		
*C*_max⁡_ (*µ*g/mL)	2.40 ± 0.10	2.60 ± 0.23
*T*_max _ (h)	1.00 ± 0.00	2.50 ± 0.00
AUC (*µ*g · h/mL)	34.5 ± 2.50	33.3 ± 1.50
SDP (h)	0.5–2.4	0.5–24

Lung		
*C*_max⁡_ (*µ*g/mL)	45.0 ± 3.00	101 ± 10.4***
*T*_max _ (h)	0.50 ± 0.00	0.50 ± 0.00
AUC (*µ*g · h/mL)	1022 ± 53.0	60.6 ± 5.00***
LDP (h)	0.5–2.5	0.5–24

PK Values are showed as the average of the 3 different assays.

***Values statistically different to the Reference group at *P* < 0.001.

*C*_max⁡_: maximum serum concentration peak, *T*_max _: time at *C*_max⁡_; AUC: area under the concentration versus time curve; SDP: Serum Detection Period; LDP: Lung Detection Period.
